# Towards Better Understanding QBism

**DOI:** 10.1007/s10699-017-9524-0

**Published:** 2017-02-21

**Authors:** Andrei Khrennikov

**Affiliations:** International Center for Mathematical Modelling in Physics and Cognitive Sciences, Linnaeus University, 351 95 Växjö, Sweden

**Keywords:** Quantum Bayesianism, Växjö interpretation, Formula of total probability, Interference of probability, Classical Bayesian physics, Universal agent

## Abstract

Recently I posted a paper entitled “External observer reflections on QBism”. As any external observer, I was not able to reflect all features of QBism properly. The comments I received from one of QBism’s creators, C. A. Fuchs, were very valuable to me in better understanding the views of QBists. Some of QBism’s features are very delicate and extracting them from articles of QBists is not a simple task. Therefore, I hope that the second portion of my reflections on QBism (or, strictly speaking, my reflections on Fuchs reflections on my earlier reflections) might be interesting and useful for other experts in quantum foundations and quantum information theory (especially, taking into account my previous aggressively anti-QBism position). In the present paper I correct some of my earlier posted critical comments on QBism. At the same time, other critical comments gained new validation through my recent deeper understanding of QBists views on a number of problems.

## Introduction

The main aim of this note is to represent my reflections on reflections of C. Fuchs (private comments via email) on my recent reflections on QBism (Khrennikov 2015a). However, it is useful to start with a general discussion about interpretations of quantum mechanics (QM) and QBism (Fuchs 2002a, b, 2007, 2012, 2014; Caves et al. 2002, 2007; Fuchs and Schack 2011, 2012, 2013, 2014; Fuchs et al. 2014; Mermin 2014a, b), in particular. Besides such basic interpretations as the Copenhagen, nonlocal, and many worlds interpretations, QBism will be compared often with the Växjö interpretation (Khrennikov 2002, 2004). The latter is not so well popularized. However, its use as a comparative illustration is justified by two things: (a) it was born (in 2001) as a realist answer to QBism (see Fuchs 2002a for QBism’s reply); (b) its essence (as well as in QBism) is the treatment of QM as a machinery for assignment of probabilities.

We start with the Copenhagen interpretation which is still the basic and commonly accepted interpretation. By this interpretation QM provides an epistemic description of micro-phenomena, i.e., results of observations; and, moreover, a finer description, than given by QM, is impossible. The latter statement is the completeness of QM from Bohr’s perspective (Bohr 1935). For him Bohr (1987) (see also Plotnitsky 2006, 2012) completeness of QM follows not from “no-go theorems” such as, e.g., von Neumann (1955) or Bell (1987) theorems, but rather from the existence of the indivisible quantum of action given by Planck’s constant *h*. The Copenhagen interpretation is completely consistent and it has been used in quantum physics for 80 years.

However, a large part of the quantum community is not completely satisfied by Bohr’s perspective (even those who use the Copenhagen interpretation in their daily routine). Theoreticians are not happy to recognize that their only task is to develop operational quantum structures. Albeit it is important for applications but definitely boring. Surprisingly, even some experimenters are unhappy. (What can they want besides the operational interpretation of QM?) Some of them, as, e.g., A. Zeilinger (in cooperation with C. Brukner), are looking for some fundamental information-processing principle behind QM (Zeilinger 1999; Brukner and Zeilinger 1999). Others (and it seems there are many) are not happy to study surrogates of features of quantum systems and their own measurement devices (and, lately, even their own free will). They want reality! They want to measure actual properties of quantum systems. This situation of very general dissatisfaction with the old Copenhagen perspective led to appearance of some very exotic interpretations of QM—so exotic that the fathers of QM are spinning in their graves. I mean nonlocal interpretations of QM^1^ and the many worlds interpretation.

*Quantum Bayesianism* (QBism) (Fuchs 2002a, b, 2007, 2012, 2014; Caves et al. 2002, 2007; Fuchs and Schack 2011, 2012, 2013, 2014; Fuchs et al. 2014; Mermin 2014a, b) is also considered exotic, surprisingly, even more exotic than the two aforementioned interpretations. Why? Maybe because QBism is a non-realistic interpretation? However, the Copenhagen interpretation is non-realistic as well... It seems that the main problem is that *QBism refers to the irreducible role of a mental element in decision making about the outcomes of quantum experiments.* And an average modern physicist is sure that in physics there is no place for mental elements. This position is a consequence of deep separation between the subject of physics and the subject of cognition and psychology. [We remind that in the nineteenth century and at the beginning of the twenieth century this separation was not so deep (Khrennikov 2015b).]

Can a mental element be peacefully incorporated into QM or in physics in general, for that matter? For the moment, the answer is “maybe”. On one the hand, we can mention the supporting views of Pauli (and his correspondence with Jung), Whitehead, and Wigner. On the other hand, we can again point to the rise of physical realism in the form of Bohmian mechanics, the many-worlds interpretation and the Växjö interpretation (Khrennikov 2002, 2004). Of course, all these “realisms” are quite exotic, being, respectively,: nonlocal, many-world, contextual.^2^

Roughly speaking, one can select between the operational, nonlocal, many world, contextual and subjective interpretations of QM. However, the presence of a mental element in decision making (in our case, about probabilities of experimental outcomes) is essentially less mystical than, e.g., spooky action at a distance. In particular, this argument was presented by T. Hänsch in his talk at the Växjö-2015 conference in which he explained why he accepts QBism as the most natural and useful interpretation of QM, especially in the light of the quantum information revolution.

Finally, we remark that QBism does not make man all that important. QBism only tries to remind that nature is not reduced to mechanical structures, that mental structures exist as well and their role has to be taken into account.

Initially, I held a strong anti-QBsm attitude. I was not happy with the subjective interpretation (de Finetti 2008) of quantum probabilities and treated them as objective (von Mises 1957; Kolmolgoroff 1933). And the Växjö interpretation was created as a realist (but contextual) answer to QBism (Khrennikov 2002). However, QBism and the Växjö interpretation have one important thing in common: both treat QM as a formalism for prediction of probabilities. And this is the essence of the formalism, not just its by-product. The difference is that in one case probabilities are interpreted as subjective and in another case as objective. Both interpretations recognize the fundamental role of the Born rule. This rule is the cornerstone of QM and the rest of the quantum formalism, including entanglement, is just a supplement. Moreover, both interpretations treat this rule as a *kind of quantum formula of total probability* (QFTP).

However, the Born rule based QFTPs have very different mathematical forms and interpretations which do not match each other; see Fuchs and Schack (2011, 2012, 2013, 2014) for the QBist version and Khrennikov (1999, 2001a, b, 2009) for the Växjö version.

In the Växjö approach QFTP appears as generalization of the classical formula of total probability (FTP). Mathematically the quantum analog of FTP is the additive perturbation of classical FTP, and the latter can be recovered for compatible observables. Interpretationally there is no difference from classical probability theory. QFTP can be treated as a generalization of classical Jeffrey’s probability update (PU), see Khrennikov (2015a).

In QBism the mathematical form of QFTP has nothing in common with classical FTP. It is even more important that here QFTP cannot be considered simply as a PU-rule. In Fuchs (2012) there was emphasized that QFTP (as a purely probabilistic representation of the Born rule) is “an addition to Bayesian probability, not in the sense of a supplier of some kind of more-objective probabilities, but in the sense of giving extra normative rules to guide the agent’s behavior when he interacts with the physical world.”

We also remark that, in contrast to the Växjö interpretation, QBism’s emphasis of the role of Born’s rule and placement of the new FTP in the center of quantum theory requires new mathematics, for instance, a very intricate number theory associated with the symmetric informationally complete measurements. This sets QBism apart from other interpretations. In the light of historical experience of development of physics one can expect that a new physical revolution would be based on novel mathematics. Therefore, the strong number-theoretic coupling of QBism can be treated as a sign that QBists are on a right pathway. At the same time one may think that QBists just borrowed this very advanced mathematical apparatus from number theory and that the next quantum-foundational revolution is likely to be based on a really new mathematical apparatus which is yet to be discovered (this is the viewpoint of A. Zeilinger, private communication).

Can one accept the use of mental elements in physics? My viewpoint to this problem changed crucially after reading Schrödinger’s book (Schrödinger 1989) which can be treated as an attempt of the mental structuring of thermodynamics, classical and quantum. Schrödinger advertised the Gibbs approach to thermodynamics based on the use of virtual ensembles composed of *mental copies* of a single system.^3^ However, in Schrödinger’s representation the Gibb’s construction is even more subjective than in the original Gibbs writings. Moreover, Schrödinger applied the same mental picture to derive quantum statistics. Hence, it seems that there is just one step to QBism?

Schrödinger did not claim that the mental representation approach is identical to the real physical situation. However, he advertised this approach, because it is simpler than Boltzmann “physical approach” and leads to the same answers! Why not accept QBism by a similar reason?^4^

## My Reflections on Fuchs’ Reflections on My Reflections on QBism

QBism is characterized by Fuchs and Schack (2014), pp. 3-4, as follows:

The fundamental primitive of QBism is the concept of experience. According to QBism, quantum mechanics is a theory that any agent can use to evaluate her expectations for the content of her personal experience. ... An agent’s beliefs and experiences are necessarily local to that agent. This implies that the question of nonlocality simply does not arise in QBism.

### QBism is Not a Neo-Copenhagen Interpretation

The viewpoint that QBism is a modern version of the Copenhagen interpretation is quite common. Therefore it is important to emphasize that this viewpoint is wrong. In Khrennikov (2015a) this problem was discussed and the position of QBists was illustrated by the citation from Mermin’s paper (Mermin 2014a, pp. 7–8):

A fundamental difference between QBism and any flavor of Copenhagen, is that QBism explicitly introduces each user of quantum mechanics into the story, together with the world external to that user. Since every user is different, dividing the world differently into external and internal, every application of quantum mechanics to the world must ultimately refer, if only implicitly, to a particular user. But every version of Copenhagen takes a view of the world that makes no reference to the particular user who is trying to make sense of that world.

Thus the main difference is the private agent perspective to outcomes of experiments which is absent in the Copenhagen interpretation. At the same time initially C. Fuchs was influenced deeply by Pauli’s version of the Copenhagen interpretation. However, to be consistent, QBism cannot restrict its mental component to Pauli’s “objective registering apparatus, the results of which are objectively available for anyone’s inspection.” Private agent’s experience is the cornerstone of QBism (Fuchs 2012).

### CBism or Even SBism?

In Khrennikov (2015a) emphasis was put on coupling of QBism to de Finetti’s subjective experience methodology of science (de Finetti 1989). In particular, it was claimed: “Finally we point that de Finetti was even more revolutionary than QBists, because his subjective treatment of scientific method was not restricted to the ‘special quantum world’. [T]hey were not brave enough to declare the private agent perspective for knowledge about classical world as well.”

This claim is a consequence of my lack of education. QBists were concentrated on QM simply because it has the most severe interpretational problems be solved in order to proceed successfully towards quantum information technologies. However, they recognized from the very beginning that the power of the subjective perspective approach can be used as well in classical statistical mechanics and in physics in general, moreover, even outside of physics (precisely as de Finetti stated). In particular, in 2003 C. Fuchs pointed out (see, e.g., Fuchs 2014, p. 812).

Since becoming immersed in the subject, I have found nothing more exciting than these trains of thought. For they indicate the extent to which quantum foundations research may be the tip of an iceberg—indeed, something with a potential to drastically change our worldview, even outside the realm of physics.

Thus QBism is just a part of de Finetti’s SBism (where ‘S’ is from ‘Science’). Another good source on the interrelation of QBism and CBism (the latter is about the private agent perspective for classical physics) is Mermin’s paper (Mermin 2014b). We stress that in this paper the mental dimension is especially strong.

### Born’s Rule as Generalization of the Formula of Total Probability

As was mentioned in the introduction, QBists treat Born’s rule as a kind of QFTP.

We remind that classical FTP functionally connects the probability distribution of one observable, say *A*, with the probability distribution of another observable, say *B*, by using the conditional probabilities $p(B\vert A)$:

1 $$p(b_j)= \sum _k p(a_k) p(b_j\vert a_k).$$

Now we present this formula in the framework of linear algebra. We introduce the vectors of probabilities $x^u=(x_k), x_k = p(u_k)$, where $u=a$ or *b*, and the vectors of transition probabilities $y^{b\vert a}_{j}= (y^{b\vert a}_{jk}), y^{b\vert a}_{jk}= p(b_j\vert a_k)$. Consider the bilinear form (scalar product) on the *n* dimensional real space $H_n$:

2 $$f(x,y) =\sum _k x_k y_k.$$

Then FTP can be written as

3 $$x^b_j =f(x^a, y^{b\vert a}_{j}) .$$

Thus FTP can be treated as the symmetric bilinear form restricted to the space of vectors composed of probabilities and conditional probabilities. In linear algebra terms, the bilinear form (2) provides the possibility to determine the probability distribution of one observable, *b*, on the basis of the probability distribution of another observable, *a*, with the aid of the $b\vert a$ conditional probabilities. We prove that such a bilinear form is determined uniquely under minimal assumptions:

Let (3) hold for the function $f(x, y)= \sum _{km} C_{km} x_k y_m$, where the coefficients $C_{km}\in {\mathbf {R}}$. Consider the case $a=b;$ then

4 $$y^{b\vert a}_{jm}= p^{a\vert a} (a_j\vert a_m) = \delta _{jm}.$$

Hence, we have the system of linear equations:

5 $$p_j^a= \sum _{km} C_{km} p_k^a \delta _{jm} = \sum _{k} C_{kj} p_k^a .$$

Choose now *a* such that it takes only one value $a=a_i$ for the fixed $k=i$, i.e., $p_i^a=1$. Such measurements can be called deterministic. Then

6 $$1= C_{ii} p_i^a , \hbox {hence}, \; C_{ii}=1,$$

and, for $$j\not =i$$,

7 $$0= C_{ij} p_i^a , \hbox {hence}, \; C_{ij}=0.$$

In the proof we did not appeal to the measure-theoretic argument. This proof is valid not only for the Kolmogorov measure-theoretic model (classical probability), but even for any *contextual probability model* in the spirit of the works of the author of this paper, e.g., Khrennikov (1999, 2001a, 2001b, 2002, 2004, 2009) (the Växjö model). We remark that a contextual probability model is represented as a collection of probability distributions $p^a_C$, where ${\mathcal {C}}= \{C\}$ is some collection of contexts of observations, and conditional probabilities which are given by matrices of the form ${\mathcal {P}}^{b\vert a}= (p(b_j\vert a_k))$. Such a matrix must be a *stochastic matrix*, i.e., for each $k, \sum _j p(b_j\vert a_k)= 1$. There are also some technical restrictions, and among them the constraint (4). This constraint can be considered as repeatability condition. The existence of deterministic measurements is another constraint.

Hence, we have shown that if some contextual probability model is endowed with a kind of FTP given by equality (3) with a bilinear form *f*, then this probability transformation should coincide with classical FTP. In Khrennikov (2009) it was shown that the quantum probability calculus can be represented as a contextual probability model. And we also know (Khrennikov 2009) that here the classical FTP is violated. Thus we cannot hope to preserve the bilinear form structure of transformation of probabilities given by (3). What is the most straightforward generalization of the bilinear form transformation? This is the addition of a constant term to a bilinear form. It happens that this strategy works for quantum probability, see (9).

Another way to generalize the classical FTP was chosen in the Växjö (contextual) probability model. Here the classical FTP is additively perturbed by an additional (“interference”) term. The latter contains square roots of probabilities and conditional probabilities. Thus, functionally the Växjö QFTP is more complicated than QBism’s QFTP. Another difference is that similarly to quantum mechanics the interference term contains new variables, “phases”, i.e., *this formula cannot be written solely in terms of probabilities* (Khrennikov 2009):

8 $$p_j^b = \sum _k p(a_k) p(b_j\vert a_k) + 2 \sum _{k< m} \cos \theta _{km} \sqrt{p(a_k) p(b_j\vert a_k) p(a_m) p(b_j\vert a_m)}.$$

Its main advantage is that this QFTP holds true for two arbitrary quantum observables *a* and *b*, e.g., given by POVMs. This is bi-observable representation of the Born rule. We also point to the *principle of correspondence*: if the interference term goes to zero, then classical FTP is recovered.

In contrast to QFTP (8), the QFTP of QBism is written for the special case of the *a*-observables given by so called SIC-POVMs (*symmetric informationally complete* POVMs, see “Appendix” for definition and basic features):

9 $$p(b_j) = (d+1) \sum _{i=1}^{d^2} p(a_i) p(b_j\vert a_i) - \frac{1}{d},$$

where *d* is the dimension of the state space.

Another important difference between the two QFTPs is that the dimension *d* of the “state space” is explicitly present in (9), but formula (8) is dimensionally invariant. For the moment, it is not clear at all whether the presence of the dimension parameter in the probability transformation rule is an advantage or disadvantage. Personally I think that such a transformation should not depend of *d*. However, not only QBism, but all information based interpretations of quantum mechanics are coupled to the dimension issue. But, how can one proceed in the infinite dimensional case? We remark that in (8) the sum can be infinite.

In Khrennikov (2015a) I was very critical of the “addiction” of QBists to such special POVMs—SIC POVMS. From my viewpoint, it would be natural to start with an arbitrary POVM *a*, at least. “However, for QBists the above generalization ? to start the probability update scheme with an arbitrary POVM measurement and not with the SIC-POVM ? eems to be unacceptable. They are really addicted to SIC-POVMs and completeness of information gained at the first step, information about the state, even at the price of appearance of counterfactuals,” (Khrennikov 2015a). In his comments C. Fuchs motivates the use of the SIC-POVM FTP representation of the Born rule as encoding “as much unique Hilbert space structure into the Born rule as possible.” It seems that QBists hope to obtain QM from this SIC-POVM FTP. And this is an exciting, but very challenging project!

The *SIC-POVM based FTP is the basic axiom of QBism.* What is its probabilistic meaning?

In Khrennikov (2015a) I interpreted SIC-POVM FTP as the quantum rule for PU, a kind of classical *Jeffrey’s conditioning* (Jeffrey 1987, 1992). Thus my viewpoint on SIC-POVM FTP resulted from my misunderstanding of the basic principle of QBism and exploring the analogy with the Växjö interpretation, where QFTP (so to say, two arbitrary POVMs FTP) is interpreted as the quantum analog of Jeffrey’s PU. This is a delicate issue and we start the discussion by reminding the classical PU and its generalization—Jeffrey’s PU.

### Classical Bayesian Probability Update

The probability of a hypothesis *H* conditioned on a collected data *E* is given by *Bayes’ formula*—the definition of conditional probability:

10 $$p(H\vert E) = p(H \& E)/p(E), p(E) > 0.$$

*Bayes’ Theorem* relates the “direct” probability of a hypothesis conditional on the data, $p(H\vert E)$, to the “inverse” probability of the data conditional on the hypothesis, $p(E\vert H)$.

11 $$p(H\vert E) = [p(H)/p(E)] p(E\vert H),$$

This possibility to “invert” probability $p(H\vert E)$, is based on commutativity of the operation of conjunction in Boolean logic: $H \& E= E \& H$. Of course, the definition of conditional probability by Bayes’ formula is possible only under this condition.

Subjectivists think of learning as a process of belief revision in which a prior subjective probability *p* is replaced by a posterior probability *q* that incorporates newly acquired information. This process proceeds in two stages. First, some of the subject’s probabilities are directly (and repeatedly) altered by earlier experience, intuition, memory, or some other non-inferential learning process. The second part is related to the knowledge just gained, that is, the subject “updates” her opinions to bring them into line with her newly acquired knowledge.

*Simple Conditioning.* If a person with a prior such that $0< p(E) < 1$ has a learning experience whose sole immediate effect is to raise her subjective probability for *E* to 1, then her post-learning posterior for any proposition *H* should be

12 $$p(H) = p(H\vert E).$$

Though useful as an ideal, simple conditioning is not widely applicable because it requires the learner to become absolutely certain of *E*’s truth. As R. Jeffrey has argued (Jeffrey 1987, 1992) the evidence we receive is often sufficient only to assign some probabilities to occurrence of *E*. Here the direct effect of a learning experience will be to alter the subjective probability of some proposition without raising it to 1 or lowering it to 0. Experiences of this sort are appropriately modeled by what has come to be called *Jeffrey conditioning.*

*Jeffrey Conditioning.* If a person has a learning experience whose sole immediate effect is to change her subjective probability for *E* to *p*(*E*), then her post-learning posterior for any *H* should be given by FTP:

13 $$p(H) = p(E) p(H\vert E) + (1 - p(E))p(H\vert \overline{E}),$$

where, for any proposition *F*, the symbol $\overline{F}$ denotes negation of the proposition *F*. Obviously, Jeffrey conditioning reduces to simple conditioning when $p(E) = 1$. Jeffrey conditioning can be generalized to the case of a collection of hypotheses and pieces of data represented mathematically as disjoint partitions of the space of elementary events $\Omega , (H_j)$ and $(E_i)$, where $H_i \& H_j=\emptyset , E_i \& E_j=\emptyset , i \not = j$:

14 $$p(H_j) = \sum _i p(E_i) p(H_j\vert E_i).$$

For further considerations, it is useful to represent Jeffrey’s PU in terms of two observables, *a* and *b*:  the events $E_i$ correspond to observations of the values $a_i$ of *a* and the hypotheses $H_j$ are about (possible) observations of the values $b_j$ of *b*. Then the PU rule (14) coincides with FTP (1).

In the pure subjective probability approach all probabilities in the right-hand side of (14) are treated as subjective. However, in classical PU it is quite common to use mixed subjective-objective PU. Here the probabilities $p(E_i)$ are considered as subjective, but the conditional probabilities $p(H_j\vert E_i)$ as objective. (In the observational notations they have the form $p(b_j\vert a_i).)$ The latter probabilities are collected, e.g., by using frequencies of observations of the hypotheses $H_j$ on the basis of events $E_i$. These are “structural constants” of the update. Soon we shall discuss these interpretations of PU given by (14) in the quantum framework.

### SIC-POVM FTP and Probability Update

Jeffrey’s PU can practically automatically be generalized to the quantum case by using POVMs representation of observables *a* and *b* and the definition of quantum conditional probability. There is a quantum state $\rho$. The information about it is updated as the result of an *a*-measurement, i.e., in general we do not try to reconstruct $\rho$ completely, but we are fine with knowing just the information gained from the *a*-measurement. (Of course, sometimes we can be lucky and be able to perform an informationally-complete measurement.) On the basis of this information and probabilities gained from sequential measurements, first *a* and then *b*, we make PU of probabilities for the possible values of the *b*-observable by using QFTP (8). In this way QFTP is interpreted in the Växjö framework (Khrennikov 1999, 2001a, b, 2009).^5^

In Khrennikov (2015a) this viewpoint on the QFTP, as a generalized PU, was extrapolated to QBism with subjective interpretation of probabilities as its main specialty, as well as the special SIC-POVM form of FTP, see (9).

However, as was pointed out in the introduction, such extrapolation was not justified and this is a real misunderstanding of QBism’s interpretation of the QFTP (in their SIC-POVM form). QBists do not consider SIC-POVM FTP as a generalization of classical Jeffrey’s PU. Their interpretation is more delicate (Fuchs and Schack 2012). Here I prefer to cite C. Fuchs (email correspondence):

I understand what FTP is, but I would never call it a machine for updating probabilities. When I think of “updating probabilities” the kind of apparatus that comes to mind is, for instance, an application of Bayes rule for conditionalizing, or Jeffrey’s conditionalization rule, etc. That is, “updating” is about changing probabilities upon the acquisition of new information. But the QFTP is not about updating in that sense. Rather QBism views it as a “coherence” statement in the sense of de Finetti’s Dutch book argument. That is, it is a relation between probability assignments, all defined at the same time (i.e., synchronically). It is not about the “changing of probabilities,” but about how various assignments should fit together to start with. Similarly, this is how we think of the Born Rule when viewed as a modified FTP: It as a specification for how various probabilities defined at one time should fit together. It is not about the changing of probabilities when information is acquired.

We also present another of Fuchs’ communications clarifying this viewpoint:

“[W]e think of it [the SIC-POVM FTP] as a synchronic coherence requirement (much like a Dutch book argument for the FTP, which is purely synchronic). Its role is to say that an agent should NOT let his probability assignment for the outcomes of a given experiment fly free from the assignments he would make in a hypothetical (or counterfactual) experiment involving an intermediate SIC. The assignments should be related even though not both experiments can be performed at the same time.” See also section 4 (Fuchs and Schack 2014) for detail.

The aforementioned synchronization of probabilities is purely subjective; even conditional probabilities (as the quantum state characteristics), *else QBism would be inconsistent.* It is important to remark that QBism, as any interpretation of QM, is evolving and may come in different flavors;^6^ for example, in the “old QBism” of Caves, Fuchs, and Schack the state and the conditional probabilities were treated objectively! We can refer to sections 7 and 8 of Fuchs (2002b), where arguments are made for the disavowal of the old QBism.

The most complicated point of the QBists interpretation of QFTP is the treatment of the *a*-measurement as a “counterfactual measurement”. We remark that counterfactuals are widely used in QM, e.g., in the proofs of Bell’s inequality. However, QBists have to be especially careful in appealing to counterfactuals. For me, the main problem is that while exploring the subjective agent perspective QBists do not aim towards coupling with cognitive science. (This is a general problem of the subjective probability interpretation.)

- Does a “counterfactual measurement” mean a kind of self-measurement performed in unconsciousness?
- Can we say that each decision maker uses SIC POVM measurements at unconscious level?
- Can QFTP be treated as coupling of such unconscious *a*-measurement with subsequent conscious *b*-measurement?

If the answer is “yes”, then QFTP can be interpreted as PU, connecting two levels of information processing, unconscious and conscious.

### Quantum Bayesian Agents

One of the reviewers of this paper finalized his/her report by the recommendation to emphasize the weak points of QBism. My private opinion is that the main problem of QBism is the rejection of the paradigm of the “universal quantum Bayesian agent”.

In Khrennikov (2015a) it was pointed out, “It is important that QBism uses this rule (Born’s rule) as an information constraint to determine a class of so to say ‘quantum agents’, i.e., those who ‘get tickets to the QBism performance.’ Thus private users of QM are those who know the main rule of the game.” This fits QBism completely. Fuchs writes, “The idea is that QM is something used only by a privileged class of people. Those educated in the methods of QM are able to make better decisions (because of certain basic features of nature) than those not educated in the methods of QM.”

However, the attempts (Khrennikov 2015a) to introduce into QBism, so to say, the “universal quantum Bayesian agent”, in the spirit of Brukner (2014) (who wrote about the “hypothetical agent”), are not welcome in QBism—experience has to be really private.

The private agent perspective matches well decision theory or game theory in which individuals make their own personal decisions, but such a totally individual perspective with respect to physical phenomena seems to be too exotic. I really believe that QBism would approach the status of methodology of natural science if quantum probabilities determined by Born’s rule were treated as probabilities assigned to events by such an “universal quantum Bayesian agent”. Of course, it is not clear whether such probabilities can be still considered as subjective. One analogy arises permanently in my mind, so I present it here, although it might be totally misleading.

Consider *Kolmogorov’s theory of algorithmic complexity.* Complexity of a string *x* is defined with respect to the concrete algorithm ${\mathcal {A}}$, denote this complexity by the symbol $K_{{\mathcal {A}}}(x)$. This type of complexity cannot be used to characterize the features of *x*. Each algorithm ${\mathcal {A}}$ generates its own quantity $K_{{\mathcal {A}}}(x)$. The main contribution of Kolmogorov to the theory of complexity was the proof of the existence of *universal algorithm*, denote it ${\mathcal {A}}_0$. Complexity with respect to any algorithm ${\mathcal {A}}$ can be coupled to complexity with respect to ${\mathcal {A}}_0$. Thus complexity can be simply defined as $K_{{\mathcal {A}}_0}(x)$.

We can use the following metaphoric picture. There is the world of algorithms (“agents”), each of them assigns its subjective value to complexity of $x, K_{{\mathcal {A}}}(x)$. However, there is the universal algorithm (“universal agent”) assigning the complexity $K_{{\mathcal {A}}_0}(x)$ to *x*. It is natural to base the notion of complexity on the latter universal quantity.

I would metaphorically treat quantum probability as an analog of the complexity $K_{{\mathcal {A}}_0}(x)$ with respect to the universal algorithm. From my viewpoint, QBism would earn a lot if it could formalize the notion of a universal quantum Bayesian agent and to prove (in the spirit of Kolmogorov) the existence of such an agent.

However, my impression is that such a strategy to “improve” QBism is totally foreign to its founders, in any event to C. Fuchs and R. Schack (private communications).

At the same time we have to understand that the aforementioned problem of the private agent versus universal agent perspectives is not the problem of QBism—“quantum” is not the crucial point. This is a general problem for the subjective probability interpretation. The latter is widely used not only in decision making performed by private agents and related to their private problems (to buy a house or shares, to marry or divorce). Nowadays the Bayesian update of probability endowed with its subjective interpretation is widely used, e.g., in engineering. Here the private agent perspective is not so natural, either. Personally I am not so well educated in the subject of subjective interpretation. I could not exclude that such questions have already been studied by (classical) Bayesians. In any event QBists have to pay more attention to this problem.

## Understanding QBism

We now summarize our discussion on the distinguished and delicate features of QBism:

1. QBism is about the private experience of agents making predictions about outcomes of experiments. The sample of agents is not arbitrary. A QBist agent has to belong to a so-to-say “quantum club”, i.e., to be “quantumly educated.”
2. The paradigm of the “universal quantum Bayesian agent” is totally foreign to QBism.
3. QBism is a natural next step from exploring mental structures in statistical physics and thermodynamics—technique of calculation of probabilities based on invention of virtual ensembles (Gibbs, Schrödinger, Jaynes). The use of such ensembles composed of mental copies of a single system naturally leads to the subjective interpretation of probabilities.
4. This matches well the scientific methodology presented by de Finettei in his great pamphlet “Probabilismo” (de Finetti 1989). According to this methodology science is about our private experiences. QBists understand well that QBism is a part of so-to-say SBism, where “S” is for science. In particular, CBism (where “C” from classical physics) was discussed in very great detail by N. D. Mermin. C. Fuchs started his pathway to QBism from subjective treatment of classical thermodynamics in the spirit of 1).^7^
5. QBism is a local interpretation of QM.
6. Is QBism a non-realist interpretation of QM? It is a complicated philosophic issue. It seems that for subjectivists (both classical, as de Finetti, and quantum) the reality is constructed from our private experiences. From this viewpoint they are realists.
7. QBism is not a version of the Copenhagen interpretation, though rooted in it.
8. According to QBism, the quantum formalism is a machinery for consistent assignment of subjective probabilities for the outputs of possible experiments.
9. This probability synchronization machinery cannot be simply treated as a kind of probability update machinery.^8^
10. The Born rule is treated as the main axiom of QM, other axioms are just supplemental.
11. This rule is represented in the form of generalized law of total probability SIC POVM FTP.^9^
12. The use of SIC POVMs is crucial, since QBists hope that the SIC POVM FTP encodes all basic features of QM.
13. For consistency of QBism all probabilities in the SIC POVM FTP have to be interpreted as subjective probabilities, even conditional probabilities $p(b_j\vert a_i)$.^10^

Finally, commenting upon 4) we remark that Fuchs was strongly influenced by the works (Jaynes 1957) and his arguments for a subjective view of classical statistical mechanics and thermodynamics were paramount in QBism from the very beginning. Already in a 19 July 1996 letter to Sam Braunstein, Fuchs wrote (Fuchs 2011, p. 443):

[Y]ou really got me interested in the old [Cox argument] again. I noticed in this version of the book that Jaynes makes some points about how there are still quite a few questions about how to set priors when you don’t even know how many outcomes there are to a given experiment, i.e., you don’t even know the cardinality of your sample space. That, it seems to me, has something of the flavor of quantum mechanics where you have an extra freedom not even imagined in classical probability. The states of knowledge are now quantum states instead of probability distributions; and one reason for this is that the sample space is not fixed—any POVM corresponds to a valid question of the system. The number of outcomes of the experiment can be as small as two or, instead, as large as you want. However I don’t think there’s anything interesting to be gained from simply trying to redo the Coxian “plausibility” argument but with complex numbers. It seems to me that it’ll more necessarily be something along the lines of: “When you ask me, “Where do all the quantum mechanical outcomes come from?” I must reply, “There is no where there.” (with apologies to [Gertrude] Stein again!) That is to say, my favorite “happy” thought is that when we know how to properly take into account the piece of prior information that “there is no where there” concerning the origin of quantum mechanical measurement outcomes, then we will be left with “plausibility spaces” that are so restricted as to be isomorphic to Hilbert spaces. But that’s just thinking my fantasies out loud.

## Appendix: Symmetric Informationally Complete Quantum Measurements

We consider one special class of atomic instruments with quantum observables given by *symmetric informationally complete* POVMs, SIC-POVMs. Here informational completeness means that the probabilities of observing the various outcomes (given by Born’s rule) entirely determine any quantum state $\rho$ being measured. This requires $d^2$ linearly independent operators for the state space of the dimension *d*.

The simplest definition is that a SIC-POVM is determined by a system of $d^2$ normalized vectors $(\phi _i)$ (they are not orthogonal) such that

15 $$\vert \langle \phi _i \vert \phi _j\rangle \vert ^2 = \frac{1}{d+1}, i \not =j.$$

The elements of the corresponding SIC-POVM $(E_i)$ are subnormalized projectors $E_i = \frac{1}{d} \Pi _i$, where $\Pi _i$ is the orthogonal projector on $\phi _i$. The elements of SIC-POVM $E_i$ determine corresponding quantum operations (atomic instruments).

Symmetry, the characteristic property of SIC-POVMs, implies that the inner product in the space of operators (or $d\times d$ matrices) given by the trace is constant, i.e.,

$$\mathrm{Tr} E_i E_j = \mathrm{const}= \frac{1}{d^2(d+1)} ,\quad i \not =j.$$

Using this equality it is easy to obtain the following representation of an arbitrary density operator $\rho$:

16 $$\rho = \sum _i \left( (d+1) p(i) - \frac{1}{d}\right) \Pi _i$$

where $p(i)= \mathrm{Tr} E_i \rho$ is the probability to obtain the result *i* for the measurement presented by the SIC-POVM $(E_i)$.

This SIC-POVM based representation of a density operator $\rho$ plays crucial role in Quantum Bayesianism (QBism), cf. with the QBist version of quantum generalization of FTP.

## Footnotes

1 In fact, there are two sorts of “quantum nonlocality”. One is the ontic nonlocality—nonlocal hidden variables (non-locality of nature as it is, when nobody observes it). It has roots in Bohmian mechanics. However, an interesting and not trivial fact is that the majority of people speaking about quantum nonlocality have in mind the epistemic nonlocality (nonlocality of measured data encoded in quantum correlations). For example, this viewpoint was presented by A. Aspect in his talk at the conference “Foundations of Probability and Physics 3”, Växjö-2005, see also his paper Aspect (2002). They say: there exists “spooky action at a distance”; this is the exhibition of “quantum nonlocality”. The latter viewpoint has to be treated as a new interpretation of QM—the *quantum nonlocality interpretation*, although people presenting such a nonlocal viewpoint consider themselves as followers of the Copenhagen interpretation. However, Bohr, Heisenberg, Pauli, Landau, and Fock definitely would not support the nonlocal interpretation of QM.

2 In the Växjö interpretation (Khrennikov 2009) contextuality is treated more generally than in modern discussions on contextuality of QM. Context is a complex of experimental physical conditions for measurement of some observable(s), cf. with the approach developed by Grangier (2002, 2005).

3 I used this book to adapt the quantum formalism to applications in social science, a model of social laser (Khrennikov 2015c).

4 And I can do this without giving up the Växjö approach, which can be considered as the quantum analog of the Boltzmann physical approach. Schrödinger did not say that Boltzmann was wrong ... Another reason for my recent movement towards QBism is my active research in applications of quantum probability to cognitive psychology (Khrennikov 2015b). Here we need an interpretation of QM which is free from spooky action at a distance and other quantum exotics. I used the Växjö interpretation, but the realist contextual modeling of cognition is too big a challenge. Therefore, it is very pragmatic to use QBism (Haven and Khrennikov 2009). We have decision makers, they assign subjective probabilities. Moreover, the latter are widely used in decision making in various areas, such as engineering, military actions, politics, economics. Engineers use subjective probability. Why as well physicists cannot?

5 Here all probabilities are interpreted objectively. However, there is nothing wrong to proceed with subjective probabilities, especially in the framework of subjective-objective PU. In the very important case of the observable *a* of von Neumann-Lüders type with nondegenerate spectrum, the conditional (or, better to say, transition) probabilities $p(b_j\vert a_i)$ do not depend on the state $\rho$. It is natural to consider them as objectively determined “structural constants”.

6 This is not a unique feature of QBism. The same can be said about, e.g., the Copenhagen interpretation. A. Plotnitsky even invented the terminology “interpretation in the spirit of Copenhagen” (Plotnitsky 2006, 2012).

7 Concentration on QBism is explained by a real necessity to solve the interpretational problems of QM in the light of the quantum information revolution.

8 This is the main difference between QBism and the Växjö interpretation of QM (more fundamental than the difference in the interpretation of probabilities).

9 This quantum analog of FTP differs crucially from the Växjö version of quantum FTP (which is an additive perturbation of classical FTP).

10 Since SIC POVMs are based on one dimensional projectors, the corresponding conditional probabilities do not depend the initial quantum state $\rho$. Hence, it is very attractive to treat them as objectively determined (as features of observables) structure constants. However, “modern QBists” would not accept such an interpretation.
